# Effect of serum albumin on cardiac-related events in end-stage kidney disease with mitral regurgitation initial hemodialysis

**DOI:** 10.1097/MD.0000000000049019

**Published:** 2026-05-29

**Authors:** Xuechun Wang, Yadan Luo, Xiaofang Tian, Liying Yuan, Xin Chen, Ling Liu, Yongli Wang, Tong Chen

**Affiliations:** a Department of Nephrology, The First People’s Hospital of Zunyi (The Third Affiliated Hospital of Zunyi Medical University), Zunyi, Guizhou, China; b Department of Burns and Plastic Surgery, Affiliated Hospital of Zunyi Medical University, Zunyi, Guizhou, China.

**Keywords:** cardiac-related events, end-stage kidney disease (ESKD), hemodialysis, mitral regurgitation, serum albumin

## Abstract

Mitral regurgitation (MR) and hypoalbuminemia are prevalent in patients with end-stage kidney disease who are initiating hemodialysis (HD) and are associated with adverse cardio-vascular outcomes. However, the combined impact of serum albumin levels and MR severity on cardiac prognosis in this population remains unclear. This study aimed to investigate the influence of serum albumin level on cardiac-related outcomes in incident HD with MR. This single-center retrospective study enrolled 217 patients with ESRD patients who initiate maintenance HD at the First People’s Hospital of Zunyi between March 2020 and February 2025. Patients were categorized into the none/mild MR (MR‐ group, n = 158) and moderate/severe MR (MR+ group, n = 59) groups based on MR severity, according to transthoracic echocardiography data. The competitive risk model and the Fine-Gray test were used to assess the impact of MR severity and serum albumin level on cardiac-related events. Subgroup and interaction analyses were performed to evaluate the impact of different factors on endpoint events. The prevalence of moderate/severe MR was 27.2%. The median follow-up duration was 20.7 months. Multivariate competing risk model analysis revealed that moderate/severe MR was an independent risk factor for cardiac-related events (subdistribution hazard ratio 2.188, 95% confidence interval 1.187–4.034; *P* = .012). Concurrently, higher serum albumin levels were an independent protective factor against cardiac-related events (per 1 g/L increase: subdistribution hazard ratio 0.914, 95% confidence interval, 0.871–0.959; *P* < .001). Subgroup analysis showed that the association between moderate/severe MR and increased cardiac event risk was stronger in patients with low serum albumin level (<35 g/L) (*P* interaction < .001), indicating a significant synergistic effect between low albumin levels and moderate/severe MR in increasing cardiac risk. Both moderate/severe MR and low serum albumin level are independent risk factors for cardiac events in patients initiating HD. Critically, low serum albumin levels significantly potentiate the adverse cardiac risk associated with moderate/severe MR. Interventions targeting serum albumin levels may improve cardiovascular outcomes in this high-risk subgroup.

## 1. Introduction

Chronic kidney disease (CKD) is an increasingly severe global health problem, affecting over 10% of the global population (approximately 850 million people).^[1,2]^ The condition progressively advances to end-stage kidney disease (ESKD), ultimately requiring renal replacement therapy. Hemodialysis (HD) is the primary treatment modality. In ESKD patients undergoing HD, the risk of cardio-vascular disease is significantly higher, affecting over two-thirds of this population, than that in the general population.^[3]^ Valvular heart diseases, such as mitral regurgitation (MR), have a high prevalence in the ESKD population.^[4]^ Studies^[5–8]^ indicate that the prevalence of MR in patients with CKD can reach 43% to 73.4%, and its prevalence, particularly moderate/severe regurgitation, progressively increases with declining glomerular filtration rate. The presence of moderate/severe MR in CKD patients is associated with adverse outcomes, including the development of atrial fibrillation (AF), increased risk of hospitalization for heart failure, and all-cause mortality.^[7–10]^

Serum albumin level is a crucial indicator of nutritional status and inflammation. Several studies have demonstrated that low serum albumin levels are correlated with a higher risk of adverse cardiovascular outcomes and both short- and long-term mortality.^[11–14]^ Due to malnutrition, inflammation, and proteinuria, serum albumin in ESKD patients is usually low, and is independently associated with cardiovascular risk and mortality in HD patients.^[15–18]^

It has been shown that low serum albumin can reduce the rate of survival in heart failure patients with secondary MR.^[19]^ However, the impact of serum albumin on cardiac-related outcomes in ESKD incident HD patients with MR remains unexplored. This study aimed to investigate the effect of serum albumin level on cardiac-related outcomes in ESKD patients with MR initial HD. By examining this relationship, we hope to deepen our understanding of the complex factors that affect the cardio-vascular health, and offer novel insights for clinical practice and management in this vulnerable population.

## 2. Methods

### 2.1. Study population

**Figure 1.**
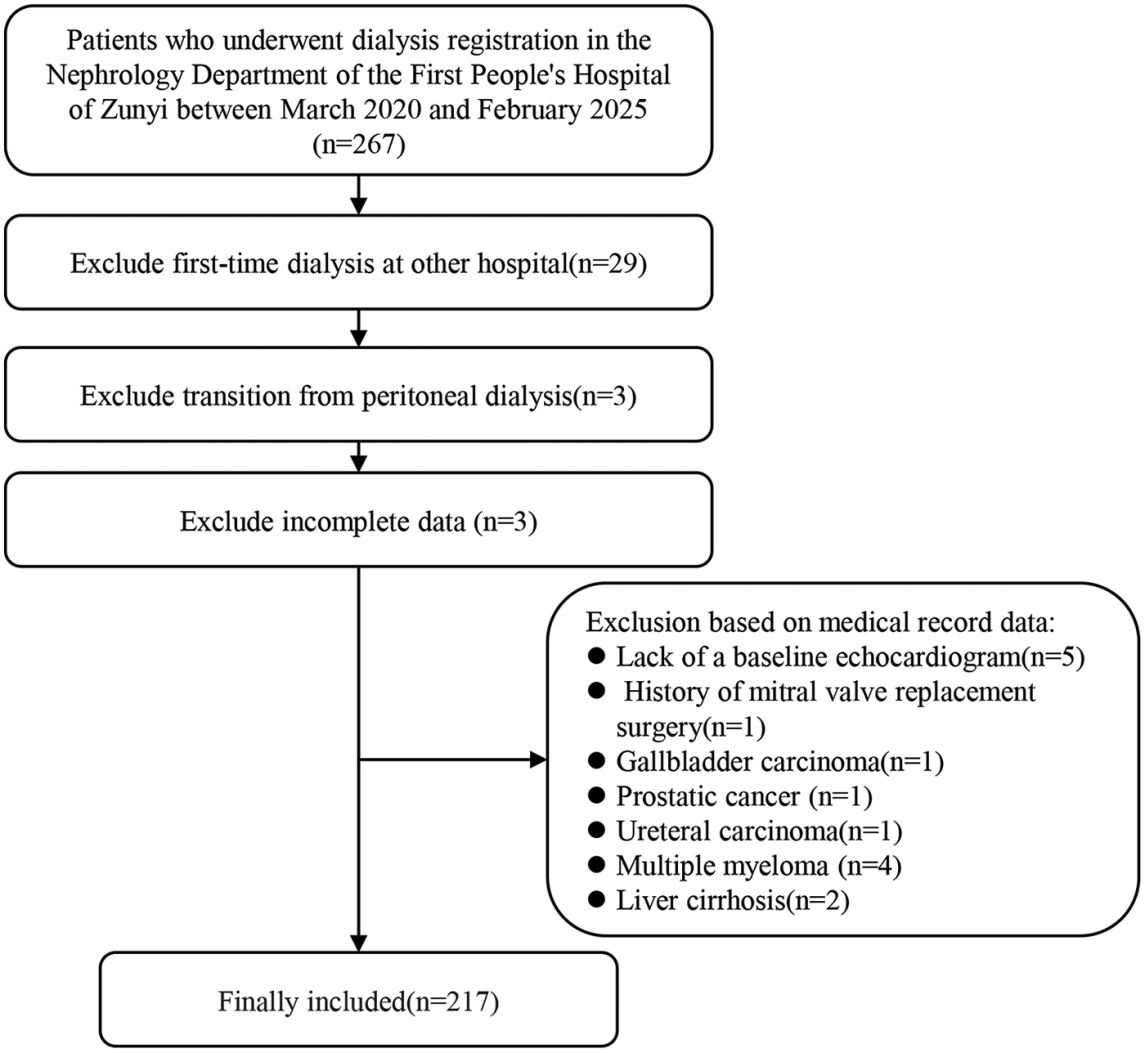
Participants flow diagram.

This single-center, retrospective study analyzed data from patients initial HD at the First People’s Hospital of Zunyi between March 2020 and February 2025. Patients aged ≥18 years who initiated maintenance HD therapy were included in our study. Patients with one of the following conditions were excluded from our study: discontinuation of HD therapy for any reason exceeding 3 months, or initiation of HD in another hospital before admission; transition from peritoneal dialysis; history of infective endocarditis, or rheumatic heart disease; history of cardiac valve surgery (specifically mitral valve replacement or mitral valve repair); history of severe heart failure with current instability; concurrent diagnosis of malignancy; concurrent diagnosis of severe liver disease; incomplete medical data or follow-up duration of <6 months. We initially screened 267 patients from the registration system. The detailed process of patient selection is illustrated in Figure 1. In brief, patients were excluded for the following reasons: first-time dialysis at other hospital (n = 29); transition from peritoneal dialysis (n = 3); incomplete data (n = 3); lack of a baseline echocardiogram (n = 5); history of mitral valve replacement surgery (n = 1); gallbladder carcinoma (n = 1); prostatic cancer (n = 1); ureteral carcinoma (n = 1); multiple myeloma (n = 4); liver cirrhosis (n = 2). A total of 217 patients were included in the final analysis. This study was approved by the Ethics Committee of the First People’s Hospital of Zunyi [approval number: Lun Shen (2024)-1-663].

### 2.2. Data collection

Patient’s demographic and clinical characteristics were collected, including age, sex, history of diabetes mellitus (DM), hypertension, coronary heart disease (CHD), chronic heart failure (CHF), and cerebrovascular disease, renal disease etiology, and body mass index. We recorded the following baseline serological parameter data before initiating dialysis: serum albumin, serum calcium (adjusted by serum albumin level), serum phosphate, parathyroid hormone, B-type natriuretic peptide (BNP), serum creatinine, urea, estimate glomerular filtration rate (eGFR), hemoglobin, total cholesterol, triglycerides, low-density lipoprotein cholesterol, high-density lipoprotein cholesterol, and C-reactive protein.

### 2.3. HD treatment

During the follow-up period, all patients were received HD 2 to 3 times per week for 4 hours every time. Additionally, they underwent hemodiafiltration once or twice and hemoperfusion once or twice per month. All patients were calculated single-pool Kt/V (spKt/V) for evaluating dialysis adequacy based on the pre- and post-dialysis urea nitrogen results every 3 months. We collected all the results and took the average as the assessment of dialysis adequacy during the follow-up period. We also collected the initial vascular access and long-term vascular access of all patients, as well as the average ultrafiltration volume (UF) and pre-dialysis systolic blood pressure (pre-HD SBP) in the first 3 month after the initiation of dialysis treatment.

### 2.4. Transthoracic echocardiography

Transthoracic echocardiography was performed in accordance with the guidelines of the American Society of Echocardiography and the European Association of Cardiovascular Imaging. Two experienced echocardiographers used a color Doppler echocardiography diagnostic instrument to conduct two-dimensional, M-mode echocardiography and Doppler flow assessment. The assessment included the size, shape, and wall thickness of all heart chambers, as well as the presence of valvular regurgitation. Both qualitative and quantitative indicators were used to diagnose MR and determine its severity. Left ventricular ejection fraction (LVEF) was used to evaluate systolic function. The ratio of early diastolic mitral valve flow velocity to late diastolic mitral valve flow velocity (E/A ratio) was used to assess diastolic function. Left ventricular mass index was calculated using the Devereux formula.

### 2.5. Endpoint events

The primary endpoint of this study was the occurrence of a cardiac-related event, including: cardiac death, the first episode of myocardial infarction, coronary artery disease requiring interventional therapy (percutaneous coronary intervention or coronary artery bypass grafting), acute heart failure (requiring hospitalization or urgent intervention), or severe arrhythmia. Severe arrhythmia is defined as the occurrence of any of the following events: persistent ventricular tachycardia or ventricular fibrillation that requires emergency electrical cardioversion or defibrillation. Symptomatic bradyarrhythmias, including symptomatic high atrioventricular block or sick sinus syndrome, and thus requiring urgent temporary or permanent pacemaker implantation. The secondary endpoint was noncardiac death.

### 2.6. Statistical methods

Statistical analyses were performed using SPSS software (version 27.0, IBM Corp., Armonk) and R software (version 4.4.3; R Foundation for Statistical Computing, Vienna, Austria). Normally distribute variables were expressed as mean ± SD, and the comparison between the 2 groups was analyzed using an independent sample *t* test. Non-normal distribution variables were expressed as median with interquartile range (*M* [*IQR*]), and the comparison between the 2 groups was analyzed using the Mann–Whitney *U* test. Categorical variables are expressed as frequency (percentage), and *Chi-square* test or *Fisher* exact probability method was used for comparison between the 2 groups. For the primary endpoint (cardiac-related events), we considered noncardiac death as a competing event. The Fine-Gray competitive risk model was used to calculate the subdistribution hazard ratio (sHR) of cardiac-related event and its 95% confidence interval (95% CI), and plot the survival curve of the cumulative incidence rate. Sensitivity analysis was used to compare the different impact of MR on the endpoint events. The traditional Cox proportional hazards model were used to analyze different endpoint events: noncardiac death as endpoint data, and noncardiac death as censored data. According to baseline age (<60 years and ≥60 years), sex (male and female), CHD, CHF, albumin level (<35 g/L and ≥35 g/L), serum calcium level (<2.1 mmol/L and ≥2.1 mmol/L), Kt/V (<1.4 and ≥1.4), UF (<2.0 L and ≥2.0 L), and pre-HD SBP (<140 mm Hg and ≥140 mm Hg), all subjects were divided into 9 subgroups. A subgroup analysis was performed to evaluate the differences between subgroups, and an interaction analysis was performed. A two-sided *P*-value <.05 was considered statistically significant for all analyses.

## 3. Results

### 3.1. Demographic characteristics and baseline data

A total of 217 patients with ESKD were included in the study. The cohort comprised 145 males (66.8%), with a median age of 59.0 (52.0–71.0) years. Comorbidities were prevalent as follows: hypertension in 178 patients (82.0%), DM in 124 patients (57.1%), coronary artery disease in 51 patients (23.5%), cerebrovascular disease in 34 patients (15.7%), and chronic heart failure in 64 patients (29.5%). The primary etiologies of ESKD were diabetic kidney disease (87 patients, 40.1%), chronic glomerulonephritis (61 patients, 28.1%), and hypertensive nephropathy (41 patients, 18.9%). Based on MR severity, 158 patients (72.8%) had none/mild MR (MR‐ group), and 59 patients (27.2%) had moderate/severe MR (MR+ group).

No statistically significant differences were observed between the 2 groups in terms of sex or age. The prevalence of concomitant CHD was significantly higher in the MR+ group than in the MR‐ group (35.6% vs 19.0%, *P* = .010). Serum albumin levels were significantly lower in the MR+ group than in the MR‐ group (34.49 ± 4.82 g/L vs 36.03 ± 4.58 g/L, *P* = .030). Low-density lipoprotein cholesterol levels (2.35 [1.94–2.81] mmol/L vs 2.08 [1.75–2.55] mmol/L, *P* = .019) and BNP levels (568.40 [249.80–1825.40] pg/mL vs 259.40 [123.60–782.90] pg/mL, *P* < .001) were significantly higher in the MR+ group. No significant differences were observed between the 2 groups in the following parameters: serum calcium, serum phosphate, calcium-phosphate product, TP, hemoglobin, high-density lipoprotein cholesterol, triglyceride, total cholesterol, C-reactive protein, serum creatinine, urea, UA, and parathyroid hormone (all *P* > .05) (Table 1).

**Table 1:** Baseline characteristics of the patients according to the baseline severity of MR.

Variables	Overall	MR‐ (n = 158)	MR+ (n = 59)	*t/z/χ^2^* value	*P* value
Male, n (%)	145 (66.8)	108 (68.4)	37 (62.7)	0.617	.432
Age, M (IQR) (yr)	59.0 (52.0, 71.0)	59.0 (51.8, 71.0)	60.00 (52.0, 72.0)	0.472	.637
Comorbidity, n (%)					
Hypertension	178 (82.0)	132 (83.5)	46 (78.0)	0.907	.341
DM	124 (57.1)	96 (60.8)	28 (47.5)	3.104	.078
CHD	51 (23.5)	30 (19.0)	21 (35.6)	6.589	.010
Cerebrovascular disease	34 (15.7)	28 (17.7)	6 (10.2)	1.854	.173
CHF	64 (29.5)	41 (25.9)	23 (39.0)	3.509	.061
Etiology of CKD, n (%)					
DKD	87 (40.1)	61 (38.6)	26 (44.1)	6.403	.350
CGN	61 (28.1)	45 (28.5)	16 (27.1)		
HKD	41 (18.9)	27 (17.1)	14 (23.7)		
Gouty nephropathy	7 (3.2)	6 (3.8)	1 (1.7)		
ADPKD	11 (5.1)	11 (7.0)	0 (0)		
Obstructive uropathy	5 (2.3)	4 (2.5)	1 (1.7)		
Others	5 (2.3)	4 (2.5)	1 (1.7)		
Adjusted calcium (mmol/L)	2.11 ± 0.22	2.11 ± 0.22	2.11 ± 0.20	0.040	.968
Phosphate (mmol/L)	1.83 ± 0.25	1.83 ± 0.25	1.84 ± 0.25	0.496	.620
Ca × Pi, M (IQR)	46.86 (43.27, 50.87)	46.74 (42.65, 50.97)	46.86 (44.36, 50.57)	0.606	.544
Alb (g/L)	35.61 ± 4.68	36.03 ± 4.58	34.49 ± 4.82	2.178	.030
TP (g/L)	63.46 ± 7.61	63.64 ± 7.76	62.99 ± 7.23	0.558	.577
HB (g/L)	88.59 ± 19.88	89.01 ± 19.27	87.44 ± 21.55	0.517	.605
TC, M (IQR) (mmol/L)	4.02 (3.27, 4.82)	3.80 (3.23, 4.69)	4.29 (3.42, 5.05)	2.044	.041
TG, M (IQR) (mmol/L)	1.56 (1.14, 2.34)	1.52 (1.10, 2.25)	1.66 (1.20, 2.41)	1.182	.237
LDL-C, M (IQR) (mmol/L)	2.16 (1.78, 2.61)	2.08 (1.75, 2.55)	2.35 (1.94, 2.81)	2.343	.019
HDL-C, M (IQR) (mmol/L)	1.04 (0.89, 1.26)	1.05 (0.88, 1.26)	1.04 (0.90, 1.25)	0.328	.743
CRP, M (IQR) (mg/L)	4.60 (1.50, 9.40)	4.85 (1.90, 9.35)	3.00 (1.10, 10.40)	1.602	.109
Scr, M (IQR) (μmol/L)	685.90 (512.50, 934.65)	698.10 (537.73, 947.80)	603.10 (446.70, 924.00)	1.541	.123
UA, M (IQR) (μmol/L)	426.10 (357.65, 513.20)	424.15 (354.35, 504.98)	439.60 (364.30, 529.00)	0.608	.544
Urea, M (IQR) (mmol/L)	23.30 (17.50, 30.70)	23.65 (17.83, 31.50)	22.30 (16.30, 29.10)	1.035	.301
eGFR (mL/min/1.73 m^2^)	6.70 (4.83, 9.44)	6.69 (4.68, 8.76)	6.95 (5.12, 11.91)	1.307	.191
PTH, M (IQR) (pg/mL)	361.70 (189.45, 514.75)	374.85 (195.98, 517.88)	272.00 (159.30, 782.90)	1.319	.187
BNP, M (IQR) (pg/mL)	295.40 (155.60, 902.40)	259.40 (123.60, 782.90)	568.40 (249.80, 1825.40)	3.680	<.001
RAASi, n (%)	65 (30.0)	43 (27.2)	22 (37.3)	2.077	.149
β-blocker, n (%)	54 (24.9)	39 (24.7)	15 (25.4)	0.013	.911

ADPKD = autosomal dominant polycystic kidney disease, Alb = serum albumin, BNP = B-type natriuretic peptide, Ca × Pi = calcium-phosphorus product, CGN = chronic glomerulonephritis, CHD = coronary heart disease, CHF = chronic heart failure, CKD = chronic kidney disease, CRP = C-reactive protein, DKD = diabetic kidney disease, DM = diabetes mellitus, eGFR = estimated glomerular filtration rate, HB = hemoglobin, HDL-C = high density lipoprotein cholesterol, HKD = hypertensive kidney disease, LDL-C = low density lipoprotein cholesterol, MR = mitral regurgitation, PTH = parathyroid hormone, RAASi = renin–angiotensin–aldosterone system inhibitors, Scr = serum creatinine, TC = total cholesterol, TG = triglyceride, TP = serum total protein, UA = uric acid.

### 3.2. Echocardiographic findings

The main pulmonary artery diameter, left atrial anteroposterior diameter, left ventricular internal dimension at end-diastole, left ventricular mass index and right atrial transverse diameter were significantly larger in the MR+ group than in the MR‐ group (all *P* < .05). The interventricular septal thickness at end-diastole and LVEF were lower in the MR+ group than in the MR‐ group (all *P* < .05) (Table 2).

**Table 2:** Comparison of echocardiographic parameters.

Variables	Overall	MR‐ (n = 158)	MR+ (n = 59)	*z/χ^2^* value	*P* value
AAoD (mm)	33.00 (31.00, 36.00)	34.00 (32.00, 36.00)	33.00 (31.00, 36.00)	0.864	.388
MPAD (mm)	22.00 (20.00, 23.00)	22.00 (20.00, 22.00)	22.00 (21.00, 24.00)	3.346	<.001
LA-ap (mm)	39.00 (36.50, 42.00)	38.00 (35.00, 41.00)	41.00 (38.00, 44.00)	4.502	<.001
LVIDd (mm)	50.00 (47.00, 55.00)	49.50 (46.75, 53.00)	54.00 (51.00, 58.00)	5.269	<.001
LVMi (g/m^2^)	120.80 (104.75, 137.50)	114.25 (101.48, 134.40)	132.00 (113.60, 144.30)	3.816	<.001
RATd (mm)	37.00 (35.00, 38.00)	36.00 (35.00, 38.00)	37.00 (36.00, 39.00)	1.976	.048
RV-ap (mm)	18.00 (17.00, 18.50)	18.00 (17.00, 18.00)	18.00 (18.00, 19.00)	1.626	.104
RVBD (mm)	36.00 (34.00,37.00)	35.00 (34.00, 37.00)	37.00 (35.00, 38.00)	2.232	.026
IVSd (mm)	10.00 (10.00, 12.00)	11.00 (10.00, 12.00)	10.00 (10.00, 12.00)	1.996	.046
LVEF (%)	60.00 (56.00, 60.00)	60.00 (58.75, 62.00)	55.00 (45.00, 60.00)	5.208	<.001
LVDD, n (%)	153 (70.5)	123 (77.8)	30 (50.8)	15.061	<.001
Tricuspid regurgitation, n (%)					
None	19 (8.8)	16 (10.1)	3 (5.1)	25.541	<.001
Mild	155 (71.4)	124 (78.5)	31 (52.5)		
Moderate	39 (18.0)	15 (9.5)	24 (40.7)		
Severe	4 (1.8)	3 (1.9)	1 (1.7)		
Aortic regurgitation, n (%)					
None	62 (28.6)	52 (32.9)	10 (16.9)	7.668	.039
Mild	119 (54.8)	82 (51.9)	37 (17.1)		
Moderate	35 (16.1)	24 (15.2)	11 (18.6)		
Severe	1 (0.5)	0 (0)	1 (1.7)		
Pulmonary arterial hypertension, n (%)					
None	179 (82.5)	140 (88.6)	39 (66.1)	14.923	.001
Mild	15 (6.9)	8 (5.1)	7 (11.9)		
Moderate	21 (9.7)	9 (5.7)	12 (20.3)		
Severe	2 (0.9)	1 (0.6)	1 (1.7)		
Pericardial effusion, n (%)					
None/trace	169 (77.9)	130 (82.3)	39 (66.1)	11.704	.005
Small	35 (16.1)	17 (10.8)	18 (30.5)		
Moderate	12 (5.5)	10 (6.3)	2 (3.4)		
Large	1 (0.5)	1 (0.6)	0 (0)		

AAoD = ascending aortic diameter, IVSd = interventricular septal thickness at end-diastole, LA-ap = left atrial anteroposterior diameter, LVDD = left ventricular diastolic dysfunction, LVEF = left ventricular ejection fraction, LVIDd = left ventricular internal dimension at end-diastole, LVMi = left ventricular mass index, MPAD = main pulmonary artery diameter, MR = mitral regurgitation, RATd = right atrial transverse diameter, RV-ap = right ventricular anteroposterior diameter, RVBD = right ventricular basal dimension.

### 3.3. Characteristics of HD treatment

Most patients who started dialysis used temporary dialysis catheters. They all switched to long-term vascular access, fistulas (including autogenous arteriovenous fistula and arteriovenous graft) and tunnel-cuffed catheters, 2 months later. There was no statistical difference in the distribution between the 2 groups (*P* > .05). There were no statistical differences in the average UF, pre-HD SBP, and spKt/V between the 2 groups (all *P* > .05) (Table 3).

**Table 3:** HD characteristics of the patients according to the baseline severity of mitral regurgitation.

Variables	Overall	MR‐ (n = 158)	MR+ (n = 59)	*z/χ^2^* value	*P* value
Initial vascular access, n (%)				2.774	.250
Fistula*	66 (30.4)	53 (33.5)	13 (19.7)		
TCC	18 (8.3)	12 (7.6)	6 (10.2)		
NCC	133 (61.3)	93 (58.9)	40 (67.8)		
Final vascular access, n (%)				2.376	.123
Fistula*	189 (87.1)	141 (89.2)	48 (81.4)		
TCC	28 (12.9)	17 (10.8)	11 (18.6)		
UF (L)	2.0 (1.7, 2.4)	2.0 (1.7, 2.4)	1.9 (1.6, 2.2)	1.436	.151
Pre-dialysis SBP (mm Hg)	142.0 (130.5, 156.0)	142.0 (132.0, 157.0)	144.0 (129.5, 155.0)	0.156	.876
spKt/V	1.42 (1.34, 1.52)	1.42 (1.35, 1.51)	1.40 (1.34, 1.53)	0.258	.797

MR = mitral regurgitation, NCC = non-cuffed catheter, pre-dialysis SBP = pre-dialysis systolic blood pressure, spKt/V = single-pool Kt/V, TCC = tunnel-cuffed catheter, UF = ultrafiltration volume.

* Fistula: including autogenous arteriovenous fistula (AVF) and arteriovenous graft (AVG).

### 3.4. Impact of mitral regurgitation on cardiac-related events

The median follow-up duration for the entire cohort was 20.7 (13.2–32.8) months until February 2025. The incidence rate of the primary endpoint (cardiac-related events) was 30.4% (66/217), and the non-cardiac death was 7.8% (17/217). There were no deaths attributable to the interventional therapy. In the MR‐ group, the median follow-up was 21.2 (14.2–33.2) months, with 37 patients (23.4%) experiencing a primary endpoint event and 15 patients (6.9%) died from noncardiac events, while in the MR+ group was 19.7 (9.6–31.1) months, with 29 patients (49.2%) experiencing a primary endpoint event and 2 patients (0.9%) died from noncardiac events (Table 4).

**Table 4:** Comparison of clinical outcomes of the 2 groups.

Variables	Overall	MR‐ (n = 158)	MR+ (n = 59)	*z/χ^2^* value	*P* value
Follow-up time (mo)	20.7 (13.2, 32.8)	21.2 (14.2, 33.2)	19.7 (9.6, 31.1)	1.615	0.106
Cardiac-related events, n (%)	66 (30.4)	37 (23.4)	29 (49.2)	13.26	0.002
Noncardiac death, n (%)	17 (7.8)	15 (6.9)	2 (0.9)		
Stroke	9	8	1	–	–
Infection	5	4	1	–	–
Gastrointestinal bleeding	1	1	0	–	–
Aortic dissection	1	1	0	–	–
Fatal trauma	1	1	0	–	–

MR = mitral regurgitation.

**Figure 2.**
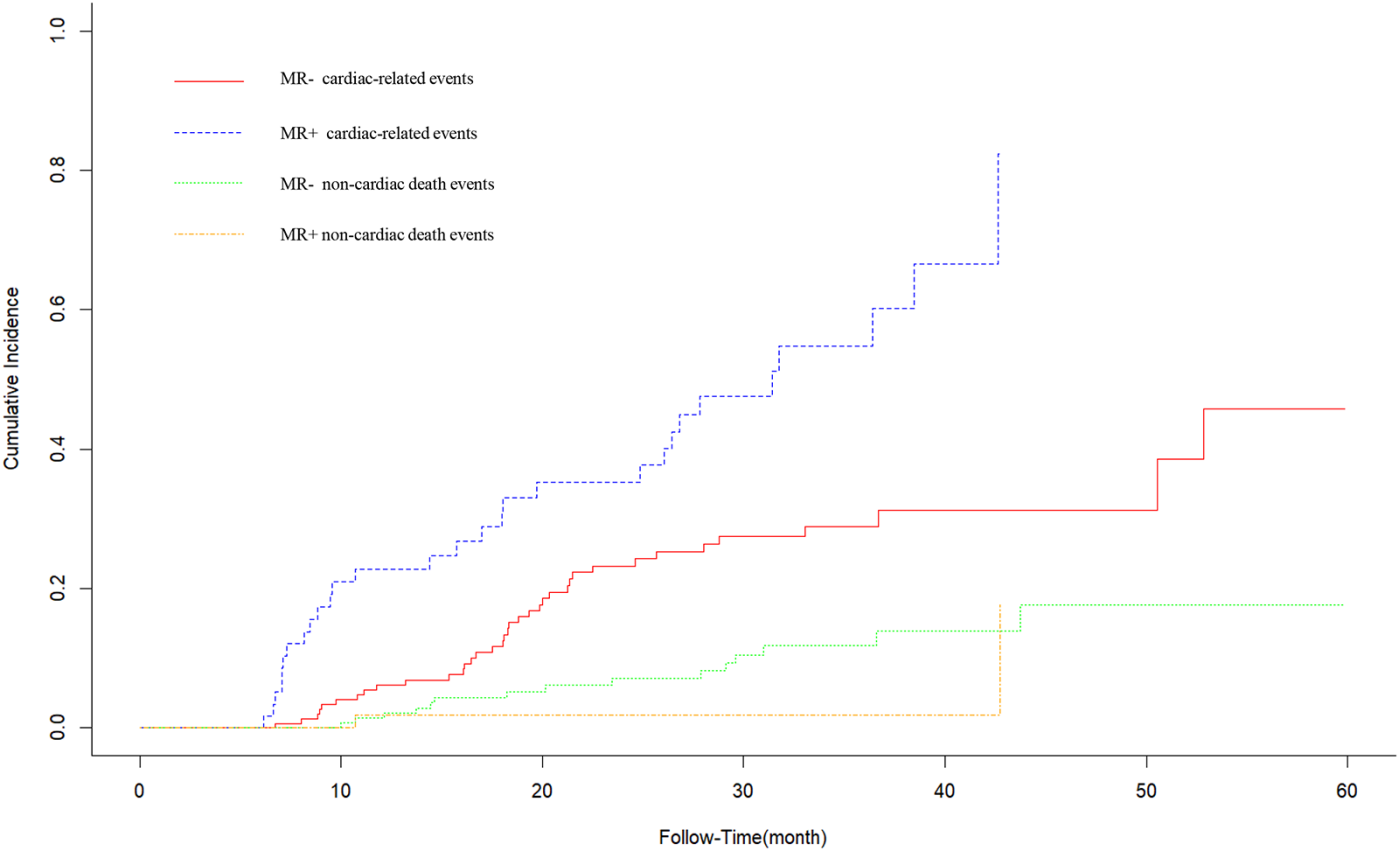
Cumulative incidence curves of cardiac-related events and cardiac death events according to MR status. The cumulative incidence of the cardiac-related events was compared across groups using the Fine-Gray model, noncardiac events as competing risks. Gray test indicated a significant difference in cumulative incidence functions by MR severity (*P* < .001). The cumulative incidence of cardiac-related events was the highest in MR+ group (blue dotted line). In contrast, the cumulative incidence of noncardiac death events was lower in MR+ group (orange dotted line). Furthermore, the incidence of cardiac-related events was higher than that of noncardiac death events in both groups. MR = mitral regurgitation.

The cumulative incidence of the primary event was compared across groups using the Fine-Gray model, accounting for competing risks. Gray test indicated a significant difference in cumulative incidence functions by MR severity (*P* < .001). As shown in Figure 2 and Table 5, the cumulative incidence of cardiac-related events was the highest in MR+ group, reaching 66.6% at 40th month. In contrast, the cumulative incidence of noncardiac death events was lower in MR+ group, consistently below 2%. Furthermore, the incidence of cardiac-related events was higher than that of noncardiac death events in both groups (Fig. 2 and Table 5).

**Table 5:** Cumulative incidence of cardiac-related events and noncardiac death according to MR groups in HD patients with ESKD (%).

Follow time (mo)	10	20	30	40	50	Stat	*P*-value
MR‐ cardiac-related events	4.0	17.7	27.4	31.2	31.2	15.287	<.001
MR+ cardiac-related events	2.1	35.2	47.6	66.6	–		
MR‐ noncardiac death events	0.7	5.2	10.5	13.9	17.6	2.028	.154
MR+ noncardiac death events	0	1.8	1.8	1.8	–		

ESKD = end-stage kidney disease, MR = mitral regurgitation.

### 3.5. Risk factors for endpoint events in HD patients with ESKD

Univariable competing risks regression analysis was used to identify the predictors of cardiac-related endpoint events in patients on HD with ESRD. The results showed that moderate/severe MR (sHR2.610, 95% CI 1.600–4.230, *P* < .001), age (sHR 1.040, 95% CI 1.020–1.060, *P* < .001), CHD (sHR 2.035, 95% CI 1.218–3.402, *P* = .007), CHF (sHR 1.890, 95% CI 1.160–3.070, *P* = .010), and eGFR (sHR 1.039, 95% CI 1.009–1.069, *P* = .009), were the risk factors. In contrast, albumin level (sHR 0.911, 95% CI 0.873–0.951, *P* < .001) and LVEF (sHR 0.955, 95% CI 0.930–0.981, *P* < .001) were protective factors (Table 6).

**Table 6:** Univariable competing risks regression analysis for cardiac events in HD patients with ESKD.

Variables	sHR (95% CI)	*P* value
MR (0 = none/mild, 1 = moderate/severe)	2.610 (1.600–4.230)	<.001
Sex (0 = female, 1 = male)	1.130 (0.372–1.910)	.640
Age (yr)	1.040 (1.020–1.060)	<.001
History of DM (0 = no, 1 = yes)	1.10 (0.677–1.780)	.700
History of hypertension (0 = no, 1 = yes)	1.370 (0.696–2.710)	.360
History of CHD (0 = no, 1 = yes)	2.035 (1.218–3.402)	.007
History of cerebrovascular disease (0 = no, 1 = yes)	0.716 (0.316–1.620)	.420
History of CHF (0 = no, 1 = yes)	1.890 (1.160–3.070)	.010
Alb (g/L)	0.911 (0.873–0.951)	<.001
Adjusted calcium (mmol/L)	1.270 (0.474–3.410)	.630
Phosphate (mmol/L)	0.644 (0.291, 1.420)	.280
Ca × Pi	0.996 (0.962–1.030)	.810
Hb (g/L)	0.999 (0.987–1.010)	.940
LDL-C (mmol/L)	0.838 (0.583–1.210)	.340
HDL-C (mmol/L)	0.482 (0.207–1.120)	.009
TG (mmol/L)	1.030 (0.843–1.250)	.790
TC (mmol/L)	0.901 (0.741–1.090)	.290
CRP (mg/L)	1.000 (0.999–1.010)	.120
Scr (μmol/L)	0.999 (0.998–1.000)	.026
UA (μmol/L)	1.000 (0.998–1.000)	.830
Urea (mmol/L)	0.980 (0.956–1.000)	.110
eGFR (mL/min/1.73 m^2^)	1.039 (1.009–1.069)	.009
PTH (pg/mL)	0.999 (0.998–1.000)	.099
BNP (pg/mL)	1.000 (1.000–1.000)	<.001
eGFR (mL/min/1.73 m^2^)	1.040 (1.020–1.060)	<.001
RAASi (0 = no, 1 = yes)	1.180 (0.698–1.980)	.540
β-blocker (0 = no, 1 = yes)	1.690 (0.977–2.860)	.051
AAoD (mm)	1.020 (0.976–1.090)	.460
MPAD (mm)	1.050 (0.982–1.130)	.150
LA-ap (mm)	1.070 (1.020–1.110)	.002
LVIDd (mm)	1.010 (0.986–1.060)	.500
LVMi (g/m^2^)	0.997 (0.989–1.010)	.510
RATd (mm)	1.030 (0.966–1.090)	.380
RV-ap (mm)	1.050 (0.992–1.120)	.088
RVBD (mm)	1.030 (0.967–1.110)	.330
IVSd (mm)	0.959 (0.806–1.140)	.640
LVEF (%)	0.955 (0.930–0.981)	<.001
LVDD (mm)	1.200 (0.682–2.110)	.530
TR (0 = none/mild, 1 = moderate/severe)	1.420 (0.844–2.390)	.190
AR (0 = none/mild, 1 = moderate/severe)	1.390 (0.815–2.370)	.230
spKT/V	0.439 (0.068–2.820)	.390
UF (L)	0.964 (0.584–1.590)	.890
Per_HD SBP (mm Hg)	1.010 (0.993–1.020)	.420
Vascular access. (0 = fistula*; 1 = TCC)	1.270 (0.620–2.590)	.520

AAoD = ascending aortic diameter, Alb = serum albumin, AR = aortic regurgitation, BNP = B-type natriuretic peptide, Ca × Pi = calcium-phosphorus product, CHD = coronary heart disease, CHF = chronic heart failure, CRP = C-reactive protein, DM = diabetes mellitus, eGFR = estimated glomerular filtration rate, ESKD = end-stage kidney disease, HB = hemoglobin, HDL-C = high density lipoprotein cholesterol, IVSd = Interventricular Septal Thickness at end-diastole, LA-ap = left atrial anteroposterior diameter, LDL-C = low density lipoprotein cholesterol, LVDD = left ventricular diastolic dysfunction, LVEF = left ventricular ejection fraction, LVIDd = left ventricular internal dimension at end-diastole, LVMi = left ventricular mass index, MPAD = main pulmonary artery diameter, MR = mitral regurgitation, pre-dialysis SBP = pre-dialysis systolic blood pressure, PTH = parathyroid hormone, RAASi = renin-angiotensin-aldosterone system inhibitors, RATd = right atrial transverse diameter, RV-ap = right ventricular anteroposterior diameter, RVBD = right ventricular basal dimension, Scr = serum creatinine, spKt/V = single-pool Kt/V, TC = total cholesterol, TCC = tunnel-cuffed catheter, TG = triglyceride, TR = tricuspid regurgitation, UA = uric acid, UF = ultrafiltration volume.

* Fistula: including autogenous arteriovenous fistula (AVF) and arteriovenous graft (AVG).

After adjusting for albumin, sex, age, CHD, CHF, adjusted calcium, blood phosphorus, BNP, eGFR, LVEF, spKt/V, UF, pre-HD SBP, and vascular access (fistula vs tunnel-cuffed catheter), the risk of cardiac-related endpoint events in the MR+ group was 2.188 times higher than that in the MR‐ group (sHR 2.188, 95% CI 1.187–4.034; *P* = .012). Meanwhile, higher albumin levels were independently associated with a significantly lower risk of cardiac-related endpoint events (sHR, 0.914; 95% CI 0.871–0.959; *P* < .001) (Table 7). The sensitivity analysis indicated that when noncardiac death events were used as the endpoint events for analysis, the severity of MR had no correlation with the occurrence of the endpoint events (HR, 1.069; 95% CI 0.580–1.971; *P* = 0830); however, when noncardiac death was used as censored data, MR+ increased the risk of cardiac-related events (HR, 1.832; 95% CI 1.001–3.352; *P* = .049) (Table 8).

**Table 7:** Multivariable competing risks regression analysis for cardiac events in HD patients with ESKD.

Variables	sHR (95% CI)	*P*-value
MR (0 = none/mild, 1 = moderate/severe)	2.188 (1.187–4.034)	.012
Alb (g/L)	0.914 (0.871–0.959)	<.001
Sex (0 = female, 1 = male)	1.228 (0.734–2.056)	.430
Age (yr)	1.043 (1.018–1.069)	<.001
History of CHD (0 = no, 1 = yes)	1.580 (0.817–3.058)	.170
History of CHF (0 = no, 1 = yes)	0.840 (0.440–1.601)	.600
Adjusted calcium (mmol/L)	1.063 (0.305–3.711)	.920
Phosphate (mmol/L)	1.119 (0.376–3.325)	.840
BNP (pg/mL)	1.000 (0.999–1.000)	.630
eGFR (mL/min/1.73 m^2^)	1.012 (0.980–1.046)	.460
LVEF (%)	0.988 (0.939–1.039)	.640
SpKt/v	0.375 (0.046–3.078)	.360
UF (L)	1.304 (0.709–2.400)	.390
Pre-HD SBP (mm Hg)	1.007 (0.992–1.022)	.380
Vascular access. (0 = fistula*; 1 = TCC)	0.489 (0.197–1.211)	.120

Alb = serum albumin, BNP = B-type natriuretic peptide, CHD = coronary heart disease, CHF = chronic heart failure, ESKD = end-stage kidney disease, LVEF = left ventricular ejection fraction, MR = mitral regurgitation, pre-HD SBP = pre-dialysis systolic blood pressure, sp-Kt/V = single-pool Kt/V, TCC = tunnel-cuffed catheter, UF = ultrafiltration volume.

* Fistula: including autogenous arteriovenous fistula (AVF) and arteriovenous graft (AVG).

**Table 8:** Multivariable Cox regression analysis for cardiac events in HD patients with ESKD.

Variables	Noncardiac death as endpoint data		Noncardiac death as censored data	
	HR (95% CI)	*P*-value	HR (95% CI)	*P*-value
MR (0 = none/mild, 1 = moderate/severe)	1.069 (0.580–1.971)	.830	1.832 (1.001–3.352)	.049
Alb (g/L)	1.037 (0.984–1.092)	.180	0.930 (0.880–0.982)	.009
Sex (0 = female, 1 = male)	0.977 (0.602–1.586)	.924	1.335 (0.762–2.340)	.312
Age (yr)	1.003 (0.986–1.021)	.728	1.039 (1.015–1.064)	.001
History of CHD (0 = no, 1 = yes)	2.100 (1.031–4.277)	.041	1.252 (0.633–2.478)	.519
History of CHF (0 = no, 1 = yes)	0.435 (0.204–0.926)	.031	0.988 (0.499–1.955)	.972
Adjusted calcium (mmol/L)	2.099 (0.626–7.036)	.230	1.230 (0.262–5.783)	.793
Phosphate (mmol/L)	1.108 (0.384–3.201)	.850	1.047 (0.266–4.128)	.948
BNP (pg/mL)	1.000 (1.000–1.000)	.837	1.000 (1.000–1.000)	.282
eGFR (mL/min/1.73 m^2^)	1.012 (0.983–1.092)	.410	0.987 (0.937–1.041)	.632
LVEF (%)	0.969 (0.930–1.010)	.140	0.988 (0.947–1.031)	.588
SpKt/v	1.230 (0.195–7.762)	.826	0.231 (0.027–2.000)	.183
UF (L)	1.008 (0.628–1.616)	.975	0.899 (0.533–1.482)	.652
Pre-HD SBP (mm Hg)	1.005 (0.992–1.018)	.428	1.008 (0.993–1.024)	.297
Vascular access. (0 = fistula*; 1 = TCC)	1.497 (1.078–2.078)	.016	1.083 (0.676–1.737)	.740

ALB = serum albumin, BNP = B-type natriuretic peptide, CHD = coronary heart disease, CHF = chronic heart failure, ESKD = end-stage kidney disease, LVEF = left ventricular ejection fraction, MR = mitral regurgitation, pre-HD SBP = pre-dialysis systolic blood pressure, spKt/V = single-pool Kt/V, TCC = tunnel-cuffed catheter, UF = ultrafiltration volume.

* Fistula: including autogenous arteriovenous fistula (AVF) and arteriovenous graft (AVG).

**Figure 3.**
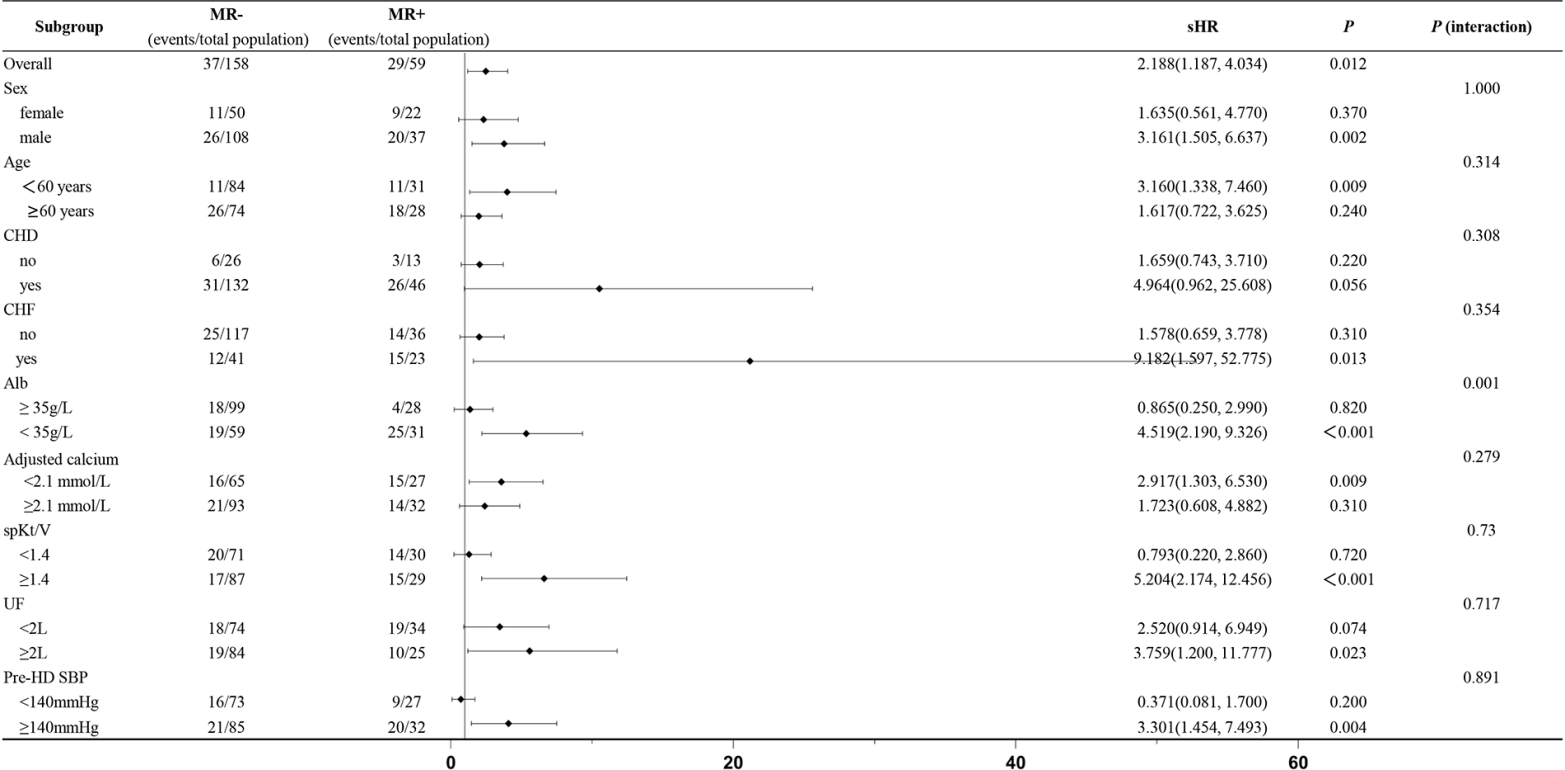
The impact of moderate/severe MR on the occurrence of cardiac-related events in each subgroup. MR = mitral regurgitation.

Forest plot results from the subgroup analysis indicated that moderate/severe MR was associated with an increased risk of cardiac-related events in male, age < 60 years, history of CHF, adjusted calcium <2.1 mmol/L, spKt/V ≥1.4, ultrafiltration volume ≥2.0 L, pre-HD SBP ≥140 mm Hg, and low serum albumin (<35 g/L). Interaction analysis revealed a statistically significant interaction between moderate/severe MR and low serum albumin levels (<35 g/L) (*P* interaction = .001). No significant interactions were observed between moderate/severe MR and any other baseline characteristics (*P* interaction > .05 for all other subgroups) (Fig. 3).

## 4. Discussion

Valvular heart disease is a common complication of CKD from ESKD. The prevalence of MR in CKD patients is significantly higher than that in non-CKD patients.^[5,20–22]^ Studies have shown that the incidence of MR in CKD patients is 1.3 to 1.8 times higher than that in non-CKD patients,^[6]^ with its prevalence, particularly moderate/severe MR, progressively increasing as glomerular filtration rate declines.^[5–8]^ The prevalence of moderate/severe MR in ESKD can reach 34.2%.^[23]^ In our study, the prevalence of moderate/severe MR among ESKD patients was 27.2%. This high prevalence is likely to be closely linked to the unique pathophysiological milieu of CKD. First, patients with CKD frequently present with comorbidities such as DM, hypertension, anemia, and fluid overload, which can induce left heart structural remodeling, including left ventricular and left atrial dilation. This dilation exerts traction on the mitral valve annulus, leading to incomplete leaflet coaptation and consequent functional MR. Moreover, following the initiation of HD for ESKD, the presence of an arteriovenous fistula increases the cardiac output. Coupled with significant fluid fluctuation during dialysis sessions, these factors can further exacerbate MR.^[5,24]^ Second, disorders of calcium and phosphate metabolism (e.g., hyperphosphatemia, secondary hyperparathyroidism, and vitamin D deficiency), chronic inflammation, and oxidative stress are highly prevalent in CKD. These abnormalities can promote fibrosis and calcification of the mitral valve annulus and leaflets, compromising valvular integrity and leading to organic MR.^[4,24]^

MR significantly increases the risks of heart failure and mortality. An Australian retrospective matched cohort study^[25]^ found that moderate/severe MR significantly increased the rates of hospitalization for heart failure and mortality risk. Similar findings were observed in the CKD population. A retrospective study of 78,059 patients who underwent echocardiography, of whom 30% (n = 23,727) had CKD, demonstrated that CKD patients with concomitant MR had significantly lower 5-year survival rates than non-CKD individuals.^[6]^ Furthermore, a cohort study including 2951 CKD patients revealed that the presence of moderate/severe MR was associated with a significantly elevated risk of developing heart failure and AF in this population.^[8]^ Consistent with these reports, our study found that ESKD patients with moderate/severe MR had a significantly higher incidence of cardiac-related events over a median follow-up period of 20.7 months, while the severity of MR has no significant impact on all-cause events.

MR induces significant alterations in cardiac hemodynamics. A portion of the ejected blood flows back into the left atrium during systole, increasing left atrial volume overload. This elevated preload can subsequently compromise blood perfusion to various tissues and organs, potentiating ischemia and hypoxia.^[7]^ Moreover, as MR progresses, left atrial pressure gradually increases. This persistent elevation not only impairs the heart’s pumping efficiency but also predisposes patients to arrhythmias, such as AF, further augmenting the cardiac workload.^[7]^ Furthermore, MR is associated with elevated levels of inflammation-related biomarkers. This pro-inflammatory state may exacerbate cardiovascular risk by promoting adverse cardiac remodeling and functional impairment.^[26]^

Hypoalbuminemia is highly prevalent in patients with CKD. Its etiology is multifactorial, potentially involving inadequate protein intake,^[18,27]^ enhanced catabolism driven by inflammation,^[28,29]^ urinary protein loss, and impaired hepatic synthesis.^[27]^ Substantial clinical evidence has confirmed serum albumin is an independent predictor of cardiovascular outcomes in patients with CKD. Early findings from the US Dialysis Outcomes and Practice Patterns Study^[28]^ identified cardiovascular death as the leading cause of mortality in HD patients. This study demonstrated that patients with baseline hypoalbuminemia had a 41% increased risk of death within the first year of dialysis initiation (adjusted HR 1.41), with an even stronger association observed during the very early dialysis period (first 120 days; adjusted HR 1.57). Furthermore, low serum albumin levels were identified as the strongest independent predictor of mortality in incident HD patients during the first 24 months of dialysis therapy. Alarmingly, a quarterly decline in serum albumin of 2 g/L was associated with a 3.07 to 3.51-fold increased mortality risk.^[16]^ Supporting the findings of this, a multicenter prospective cohort study by De Mutsert et al^[15]^ found that every 10 g/L decrease in serum albumin was associated with a 47% increased risk of all-cause mortality in HD patients, with inflammation pathways partially explaining this elevated risk. Additionally, a prospective cohort study of Chinese dialysis patients^[30]^ indicated that hypoalbuminemia mediates the increased risk of cardiovascular and all-cause mortality associated with hypokalemia, likely through mechanisms involving nutritional status and inflammatory responses. Consistent with this established evidences, the results of our study demonstrate that, after multivariable adjustment, for serum albumin increased by 1 g/L, the risk of cardiac-related events was reduced by 8.9% in ESKD patients. This finding confirms that higher serum albumin levels serve as independent protective factors against cardiac-related events in this population.

The reduction of serum albumin frequently reflects malnutrition, particularly in patients with chronic diseases, and is directly associated with an increased risk of cardiovascular events.^[31]^ Moreover, decreased serum albumin can elevate blood viscosity and reduce plasma colloid osmotic pressure, thereby increasing cardiac workload.^[18,32]^ Simultaneously, low serum albumin levels are often associated with an inflammatory state, which in turn can further contribute to cardiovascular events by depressing albumin synthesis and potentially through other inflammatory pathways.^[31,32]^

Previous studies in non-CKD populations have also demonstrated an association between serum albumin levels and prognosis in patients with MR. Findings from the COAPT trial^[19]^ indicated that among patients with heart failure and secondary MR, those with serum albumin levels of <40 g/L had significantly higher all-cause mortality. Furthermore, Shibata et al^[33]^ on patients undergoing transcatheter edge-to-edge mitral valve repair showed that postoperative improvement in serum albumin levels was independently associated with reduced all-cause mortality and could also lower the risk of heart failure hospitalization. Low serum albumin levels and MR may synergistically contribute to increased risk of cardiovascular events. This likely occurs through multiple interconnected pathways that adversely affect hemodynamics, increas cardiac workload, and potentiat inflammatory responses.

However, there are no relevant studies on the effect of serum albumin on cardiovascular outcomes in ESKD patients with MR. This study is the first to reveal the synergistic prognostic value of serum albumin and MR severity in patients in the initial HD phase. Our results demonstrate that low serum albumin levels and moderate/severe MR exert a synergistic effect, significantly increasing the risk of cardiac-related events in patients with ESKD.

### 4.1. Limitations

Our study has several limitations. First, as a single-center, retrospective study, the sample size was relatively small. Although multivariable regression models were used to adjust for known confounding factors, residual confounding due to unmeasured variables (such as the extent of vascular calcification, residual kidney function, antihypertensive regimens/doses, detailed nutritional indices, inflammatory burden, and objective volume status, and other unknown confounding factors) could potentially influence the results. Therefore, the findings should be interpreted as demonstrating adjusted associations rather than establishing causal relationships. Second, serum albumin levels are regulated by inflammation and nutritional status, and our analysis relied solely on a single baseline measurement. Consequently, its observed association with cardiovascular outcomes may be underestimated if albumin improves with nutritional or volume interventions during follow-up. Third, we collected echocardiographic data only from a single baseline. This limitation stems from our dialysis center’s practice, in which routine echocardiography is not performed in clinically stable HD patients. It is typically conducted only upon subsequent hospital admission due to complications. Moreover, the retrospective grading of MR severity in patients with fluctuating volume status may have led to some misclassification, which would also likely bias associations toward the null. In addition, due to the retrospective design, inter- and intra-observer variability for the cardiac ultrasound parameters was not formally evaluated. Fourth, the subgroup and interaction analyses conducted were exploratory in nature and were not adjusted for multiple comparisons; thus, these results should be interpreted with caution as hypothesis-generating, and borderline findings should not be overinterpreted. Given these limitations, further verification is needed through strictly designed prospective multicenter studies, combined with the dynamic changes in serum albumin levels and echocardiographic indicators.

## 5. Conclusion

MR is highly prevalent in patients with ESKD. Both serum albumin levels and moderate/severe MR were identified as independent risk factors for cardiac-related events in patients with ESKD on initial HD. Critically, these 2 factors exhibit a significant synergistic effect. Specifically, low serum albumin levels significantly exacerbated the risk of cardiac-related events in initial HD patients with concomitant moderate/severe MR. These findings suggest that interventions aimed at improving serum albumin levels may potentially reduce the risk of cardiac-related events and improve clinical outcomes in this high-risk patient population.

## Acknowledgments

The authors gratefully acknowledge the Zunyi City Science and Technology Bureau, China [Grant Number: Zunshi Kehe HZ (2025) No. 27], for funding this study.

## Author contributions

**Conceptualization:** Xuechun Wang, Xiaofang Tian, Liying Yuan.

**Data curation:** Xin Chen, Ling Liu, Yongli Wang, Tong Chen.

**Formal analysis:** Xuechun Wang, Xiaofang Tian.

**Funding acquisition:** Yadan Luo.

**Methodology:** Yadan Luo, Xiaofang Tian.

**Supervision:** Liying Yuan.

**Writing – original draft:** Xuechun Wang.

**Writing – review & editing:** Yadan Luo, Xiaofang Tian.
